# The prognosis of different types of pleural tags based on radiologic-pathologic comparison

**DOI:** 10.1186/s12885-022-09977-4

**Published:** 2022-08-25

**Authors:** Yao Meng, Jie Gao, Chongchong Wu, Mei Xie, Xidong Ma, Xuelei Zang, Jialin Song, Meng Zhou, Shikun Guo, Yemei Huang, Hengyu Deng, Hongli Li, Bo Wei, Xinying Xue

**Affiliations:** 1 Department of Thoracic Surgery, Beijing Shijitan Hospital, Capital Medical University, Beijing, China; 2 Department of Pathology, the First Medical Centre, Chinese PLA General Hospital, Beijing, China; 3 Department of Imaging, the First Medical Centre, Chinese PLA General Hospital, Beijing, China; 4 Department of Respiratory and Critical Care Medicine, the First Medical Centre, Chinese PLA General Hospital, Beijing, China; 5 Department of Laboratory Medicine, the First Medical Centre, Chinese PLA General Hospital, Beijing, China; 6 Weifang Medical University, Weifang, China; 7 School of Medical Imaging, Binzhou Medical University, Yantai, China; 8 Peking University Health Science Center, Beijing, China; 9 Capital Medical University, Beijing, China; 10 Department of Thoracic Surgery, Beijing Tiantan Hospital, Capital Medical University, Beijing, China; 11 Department of Respiratory and Critical Care Medicine, Beijing Shijitan Hospital, Capital Medical University, Beijing, China

**Keywords:** Visceral pleural invasion, Pleural tag, Computed tomography, Pathology, Prognosis

## Abstract

**Objectives:**

There are increasing numbers of studies of pleural tags (PTs). The purpose of this case series was to classify the PTs in patients with peripheral pulmonary adenocarcinoma based on radiologic-pathologic comparison and to study the prognosis.

**Methods:**

The clinical, imaging, pathological and prognostic data of 161 patients with peripheral pulmonary adenocarcinoma in three hospitals were analyzed retrospectively. We classified PTs using computed tomography (CT) for pathologic comparison.

**Results:**

According to the relationship between tumors and pleural on CT images, PTs were classified into four types: type 1, one or more linear pleural tag; type 2, one or more linear pleural tag with soft tissue component at the pleural end; type 3, one soft tissue cord-like pleural tag; type 4, directly abutting the visceral pleura, pulling or pushing the visceral pleura. In these PTs, the incidence of visceral pleural invasion (VPI) was high in type 2 (46.88%) and type 3 (56.41%) of PTs. Our prognostic analysis showed that micropapillary or solid histological subtype (HR = 5.766, 95% CI: 1.435–23.159, *P* = 0.014) and type 3 of PTs (HR = 11.058, 95% CI: 1.349–90.623, *P* = 0.025) were two independent risk factors for tumor progression.

**Conclusions:**

PT is a risk factor for poor prognosis in patients with peripheral pulmonary adenocarcinoma, the presence of which on CT images can remind us to provide patients with a more reasonable treatment.

## Introduction

Lung cancer is the primary cause of cancer-related mortality in most developed countries, with 5-year relative survival rates of 18% at present [1–3]. Currently, although surgical resection is the treatment of first choice for early stage non-small cell lung cancer (NSCLC), most patients have either locally advanced or metastatic disease, and only 20–30% of patients have potentially operable early stage disease [4]. The tumor node metastasis (TNM) staging influences the diagnosis, treatment plan and prognosis for this malignancy [1]. Visceral pleural invasion (VPI) is a significant stage descriptor in the eighth edition of the TNM staging system. Mayumi et al. [5] reported that the five-year survival rates decreased from 86% (for patients without VPI) to 62–70% (for patients with VPI). VPI is defined as the tumor that extends to the elastic layer of visceral pleura but not beyond the surface of visceral pleura [6, 7]. For parietal pleural invasion, extrapleural dissection with en-bloc resection were recommended [8], and lobectomy rather than segmentectomy was recommended in patients with VPI [9]. Therefore, accurate diagnosis of VPI is important preoperatively.

Computed tomography (CT) is currently the imaging method of first choice in the staging of lung cancer. The diagnosis of VPI on CT mainly depends on the contact of the tumor with the chest wall, mediastinum or interlobar fissure. However, simple contact of tumor and chest wall, mediastinum or interlobar fissure does not necessarily mean invasion. Pleural tags (PTs) refer to one or more linear strands extending from the nodule surface to the pleural surface due to thickening of interlobular septa of the lung. PTs on CT images may help us improve the accuracy of early diagnosis of VPI [10]. The purpose of this case series was to classify the PTs in patients with peripheral pulmonary adenocarcinoma based on radiologic-pathologic comparison and to study the prognosis.

## Materials and methods

### Patients

With approval from our institutional review board, our study was performed with exemption of informed consent. We retrospectively collected and analyzed the clinical, CT, and pathologic data in 161 patients with peripheral pulmonary adenocarcinoma. They were treated at one of three hospitals (the General Hospital of the People's Liberation Army, Affiliated Beijing Shijitan Hospital of Capital Medical University and Affiliated Hospital of Qingdao University) between 2013 and 2019. Imaging data were retrieved from the picture archiving and communication system (PACS). Tumors were characterized as peripheral pulmonary adenocarcinoma, and the diagnosis was confirmed by postoperative histopathologic examination. Sex, age, pleural invasion, the largest diameter of the tumor, pathological type, histological subtype and pathologic T stage (pT stage) of 161 patients were recorded. Tumor stage was determined according to the 2017 Union for International Cancer Control (UICC) TNM Staging (8th edition).

In addition to typical PTs on CT, only patients without other malignancies and without any postoperative adjuvant therapy were included in this study.

### CT analysis

All patients included in this study received non-contrast CT of the chest performed by a GE LightSpeed 16-Slice CT scanner (GE Healthcare, Beijing, China) or a Siemens SOMATOM Sensation 64-Slice CT Scanner (Siemens, Forchheim, Germany). The CT parameters were as follows: routine section thickness, 1.0, 1.25, or 1.5 mm; section thickness after reconstruction, 0.625–1.25 mm; filtered back projection reconstruction method; 80–120 kV; 200–280 mAs; and a B70f kernel. We used the last CT studies in the lung just before histopathologic diagnosis as the CT observation.

A thoracic radiologist with 26 years of experience in cardiopulmonary imaging and a medical student with 2 years of experience in pulmonary imaging diagnosis consistently examined the CT images of each institution by using a PACS (AGFA Healthcare, Mortsel, Belgium) (lung window width, 1500 HU; level, -500 HU) and labeled the CT images including PTs. PTs were recorded as the radiological parameter for each patient.

### Pathological analysis

All existing histopathologic slides were reviewed by one senior pathologist with 12 years of experience with pathologic diagnosis of the lung and a Master of Pathology with 3 years of experience with pathologic diagnosis.

### Statistical analysis

We used telephone follow-up, but 29 patients were lost to follow-up immediately after surgery among all the 161 patients. Data were collected and entered by using Microsoft Excel and were analyzed using SPSS, version 26.0 (IBM Statistics, Armonk, NY). Age and the largest diameter of the tumor were expressed as means ± standard deviations with ranges. Sex, diagnosis of VPI, pathologic type, histological subtype, pT stage and CT features are expressed as frequencies and percentages. Progression-free survival (PFS) was estimated using the Kaplan–Meier method, and differences in survival rates were determined by log-rank test. Variables with *p* value < 0.15 were included in the Cox-proportional hazards model for multivariable survival analysis to evaluate independent risk factors affecting the prognosis of patients. A *p* value < 0.05 was considered statistically significant.

## Results

### Clinical characteristics

**Fig. 1:**
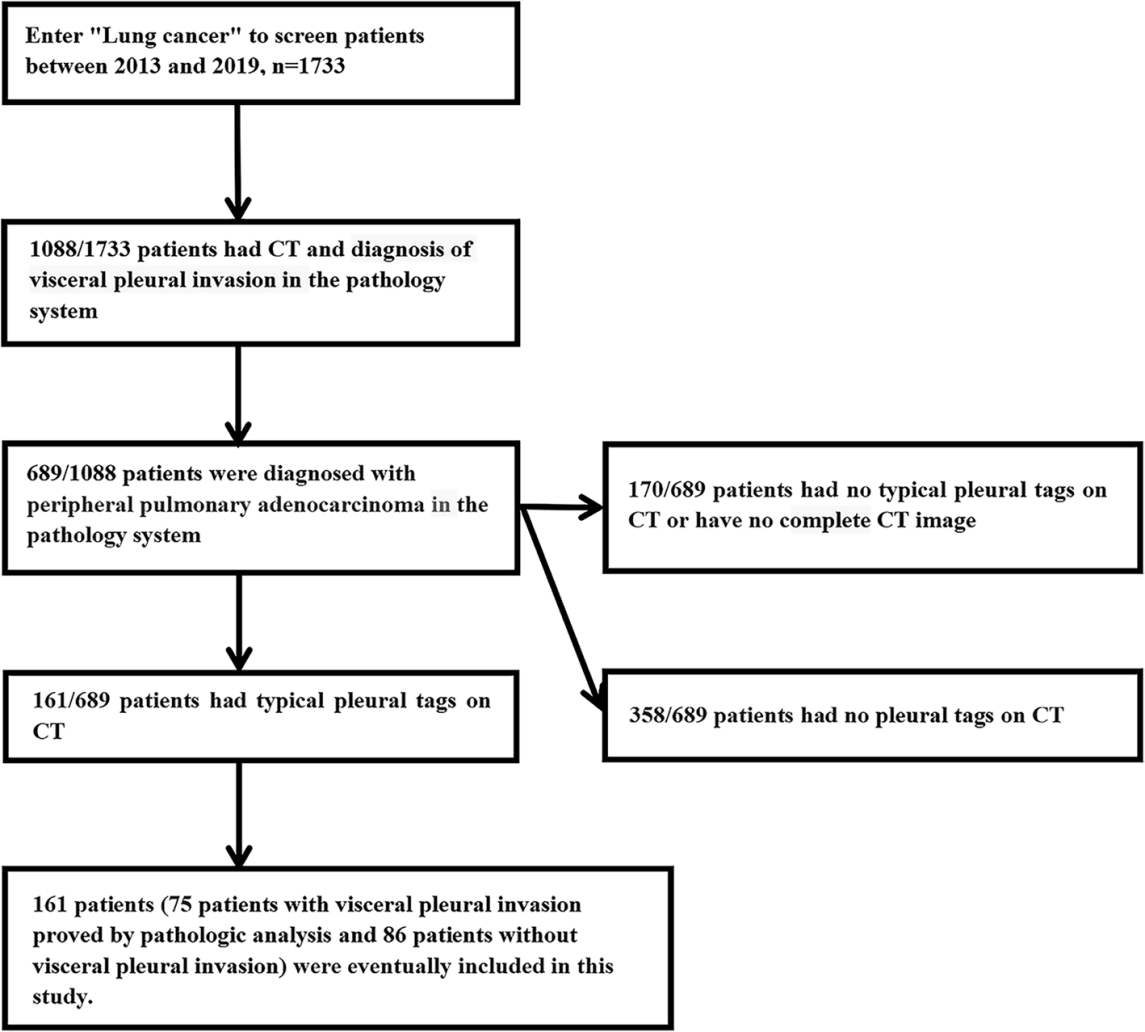
Patient inclusion flowchart shows the number of patients, evaluation of the imaging studies and pathologic analysis

Figure 1 shows the patient inclusion flowchart. A total of 161 patients (mean age, 59.67 years ± 10.44; age range, 27–84 years), with peripheral pulmonary adenocarcinoma were included (Table 1). Sixty-two (38.50%) of the 161 patients were mean (mean age, 60. 73 years ± 10.00; age range, 33–84 years), and 99 (61.50%) were women (mean age, 59.01 years ± 10.71; age range, 27–84 years). Seventy-five patients (46.58%) of the 161 patients were diagnosed with VPI and eighty-six patients (53.42%) were diagnosed without VPI. The largest diameter of the 161 patients’ tumor was 2.32 cm ± 1.33, and the diameter ranged from 0.5-11 cm.

**Table 1:** Clinical characteristics of patients with peripheral pulmonary adenocarcinoma

Characteristic	No. of Patients	Datum
Age of all 161 patients (y)	161	59.67 ± 10.44(27–84)
Male		60.73 ± 10.00(33–84)
Female		59.01 ± 10.71(27–84)
Sex of all 161 patients		
Male	62	38.50%
Female	99	61.50%
VPI		
Yes	75	46.58%
No	86	53.42%
Largest diameter of 161 patients’ tumor	161	2.32 ± 1.33(0.5–11)
Pathological type of 161 patients		
MIA	3	1.86%
INMA	153	95.03%
IMA	5	3.11%
Histological subtype of 161 Patients		
LPA	29	18.01%
APA	78	48.45%
MPA or SPA	14	8.70%
PPA	16	9.94%
Not available	24	14.90%
pT stage		
T1	76	47.21%
T2	81	50.31%
T3 ~ T4	4	2.48%

*Note*. Data are means ± standard deviations or percentages. Data in parentheses are ranges

*pT stage* Pathologic T stage, *IMA* Invasive mucinous adenocarcinoma, *MIA* Minimally invasive adenocarcinoma, *INMA* Invasive non-mucinous adenocarcinoma, *LPA* Lepidic predominant adenocarcinoma, *APA* Acinar predominant adenocarcinoma, *MPA* Micropapillary predominant adenocarcinoma, *PPA* Papillary predominant adenocarcinoma, *SPA* Solid predominant adenocarcinoma, *Not available* The histological subtype was not clearly defined

Three patients (1.86%) of the 161 patients were diagnosed with minimally invasive adenocarcinoma (MIA) in the pathology system, one hundred and fifty-three patients (95.03%) were diagnosed with invasive non-mucinous adenocarcinoma (INMA) and five patients (3.11%) were diagnosed with invasive mucinous adenocarcinoma (IMA). Twenty-nine patients (18.01%) of the 161 patients were diagnosed with lepidic predominant adenocarcinoma (LPA) in the histology system, seventy-eight patients (48.45%) were diagnosed with acinar predominant adenocarcinoma (APA), sixteen patients (9.94%) were diagnosed with papillary predominant adenocarcinoma (PPA), fourteen patients (8.70%) were diagnosed with micropapillary predominant adenocarcinoma (MPA) or solid predominant adenocarcinoma (SPA) and for twenty-four patients (14.90%) data were not available.

Seventy-six patients (47.21%) were diagnosed with pT1 in the pT stage system, eighty-one patients (50.31%) were diagnosed with pT2, and four patients (2.48%) were diagnosed with pT3 or pT4.

### CT characteristics

**Fig. 2:**
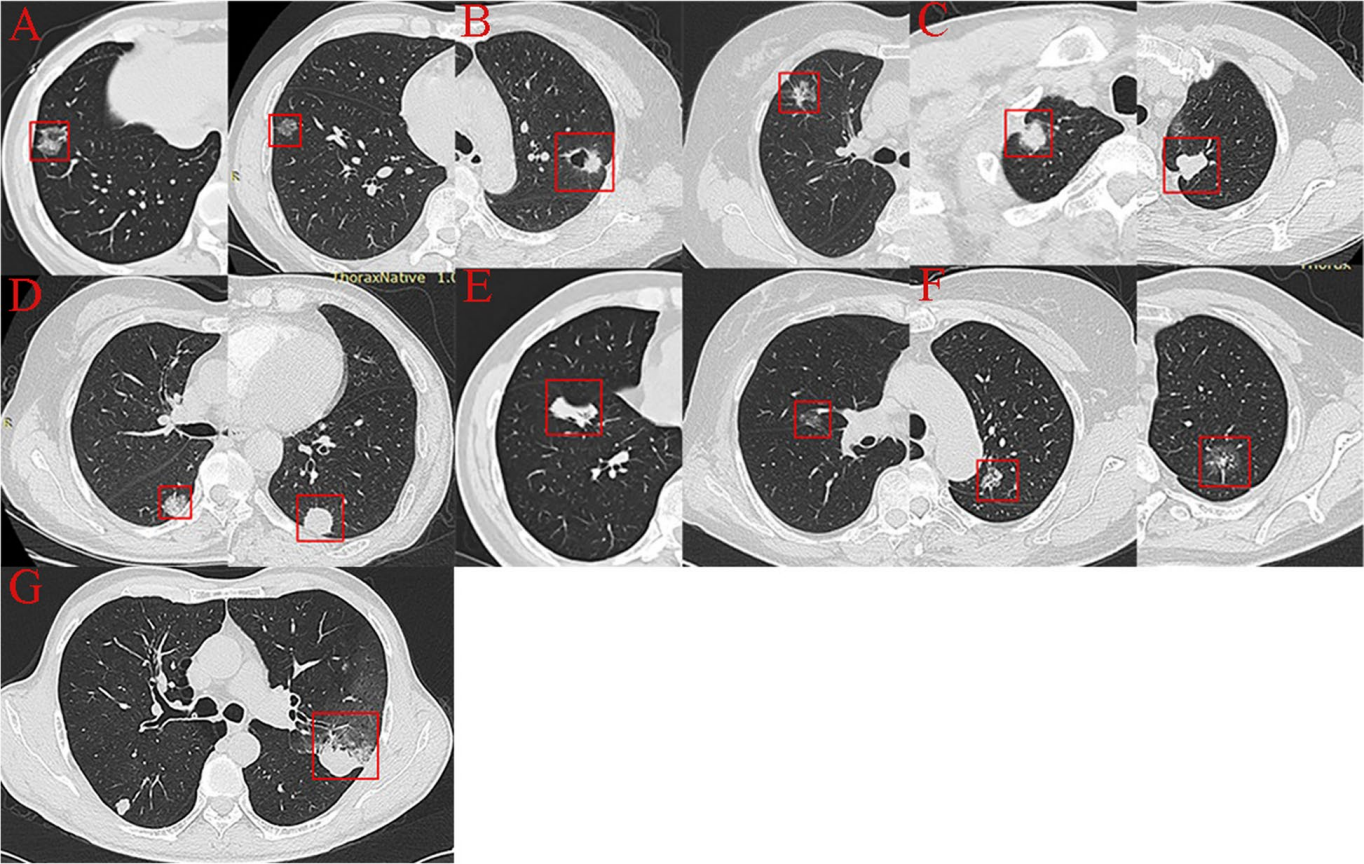
Pleural tags on CT: Type 1 (**A**, **F**), Type 2 (**B**), Type 3 (**C**), Type 4 (**D**, **E**, **G**). The first image in each group had the pleural tag with VPI confirmed pathologically, and the second had not. There was only one image in group G (type 4 of pleural tags with VPI)

Figure 2 shows the CT imaging features of the peripheral pulmonary adenocarcinoma consistently determined by a thoracic radiologist and a medical student with experience in pulmonary imaging diagnosis. The CT features were classified into four types: type 1, one or more linear pleural tag; type 2, one or more linear pleural tag with soft tissue component at the pleural end; type 3, one soft tissue cord-like pleural tag; type 4, directly abutting the visceral pleura, pulling or pushing the visceral pleura.

Twenty-four patients (14.91%; nine patients [37.50%] with pleural invasion proved by pathologic analysis) of the 161 patients had type 1 of PTs; thirty-two patients (19.88%; fifteen patients [46.88%] with pleural invasion) had type 2 of PTs; thirty-nine patients (24.22%; twenty-two patients [56.41%] with pleural invasion) had type 3 of PTs and sixty-six patients (40.99%; twenty-nine patients [43.94%] with pleural invasion) had type 4 of PTs (Table 2).

**Table 2:** CT Characteristics in Patients with Peripheral Pulmonary Adenocarcinoma

CT Characteristics^a^	VPI n (%)	non-VPI n (%)	datum
Type1	9 (37.50%)	15(62.50%)	14.91%
Type2	15 (46.88%)	17(53.12%)	19.88%
Type3	22 (56.41%)	17(43.59%)	24.22%
Type4	29 (43.94%)	37(56.06%)	40.99%

*Note*. Data are the number of patients or percentages. Data in parentheses are percentages

*PTs * Pleural tags

^a^ Based on evaluation of the last chest CT study performed before histopathologic diagnosis

### Pathology

There were 161 patients with a definitive histopathologic diagnosis. Pathologic findings confirmed that 75 (46.58%) of the 161 patients were diagnosed with VPI, and 86 patients (53.42%) were diagnosed without VPI.

**Fig. 3:**
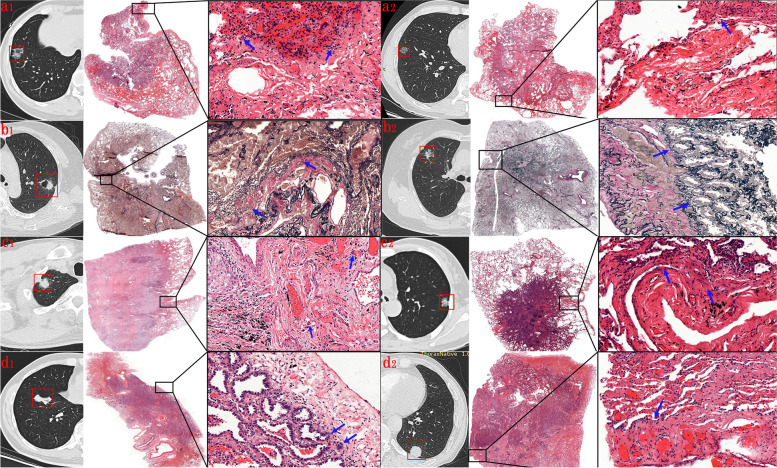
Pleural tags and corresponding hematoxylin–eosin-stained or elastica van Gieson stained histologic findings. (Original magnification, × 20.) At the arrow: (a1), (c1), (d1) tumor cells infiltrated the pleura; (a2), (c2) tumor cells were localized under the subpleural; (b1) tumor cells were observed to penetrate the elastic fibrous boundary of the pleura and infiltrate into the pleura; (b2) tumor cells did not break through the elastic layer of the pleura. (d2) Although the tumor was attached to the pleura on CT, it was pathologically confirmed to be pleural thickening and no tumor cell infiltration

After surgery, we correlated the imaging findings with the pathologic findings. According to the characteristics of the CT images, under the × 20 magnification of hematoxylin–eosin staining, type 1 of PTs (the linear PTs) was formed by the contraction of reactive proliferative fibrous tissue in the tumor. This was done by pulling the pleura to make it parallel, concave, and close to each other or the fibrous hyperplasia, and the thickening of interlobular septa, along which carcinoma cells or inflammatory cells infiltrated. Type 2 of PTs included changes in linear PTs and terminal triangular pleural indentation. Type 3 of PTs were caused by the proliferative fibrous tissue in the tumor, which contracted and pulled pleura to form a V-shape shadow or caused compressive atelectasis to form a cord-like soft tissue shadow. Type 4 of PTs showed tumor tissue attached to normal or thickened visceral pleura (Fig. 3).

In patients pathologically diagnosed with VPI, tumor cells were observed to penetrate the elastic fibrous boundary of the pleura and infiltrate into the pleura under the 20 × microscopic scale of Elastica van Gieson staining. In patients diagnosed without VPI, the tumor cells did not break through the elastic layer of the pleura (Fig. 3).

### Prognosis

Patients in this study were followed up from 2 to 77 months after surgery. Among the 132 patients included, 50 were males and 82 were females, aged 27–84 years, with a median age of 60 years. There were 32 patients with tumor progression (19 with new malignant nodules or distant metastasis, 13 died of lung cancer). Continuous variables, including age and tumor diameter, were transformed into categorical variables.

**Fig. 4:**
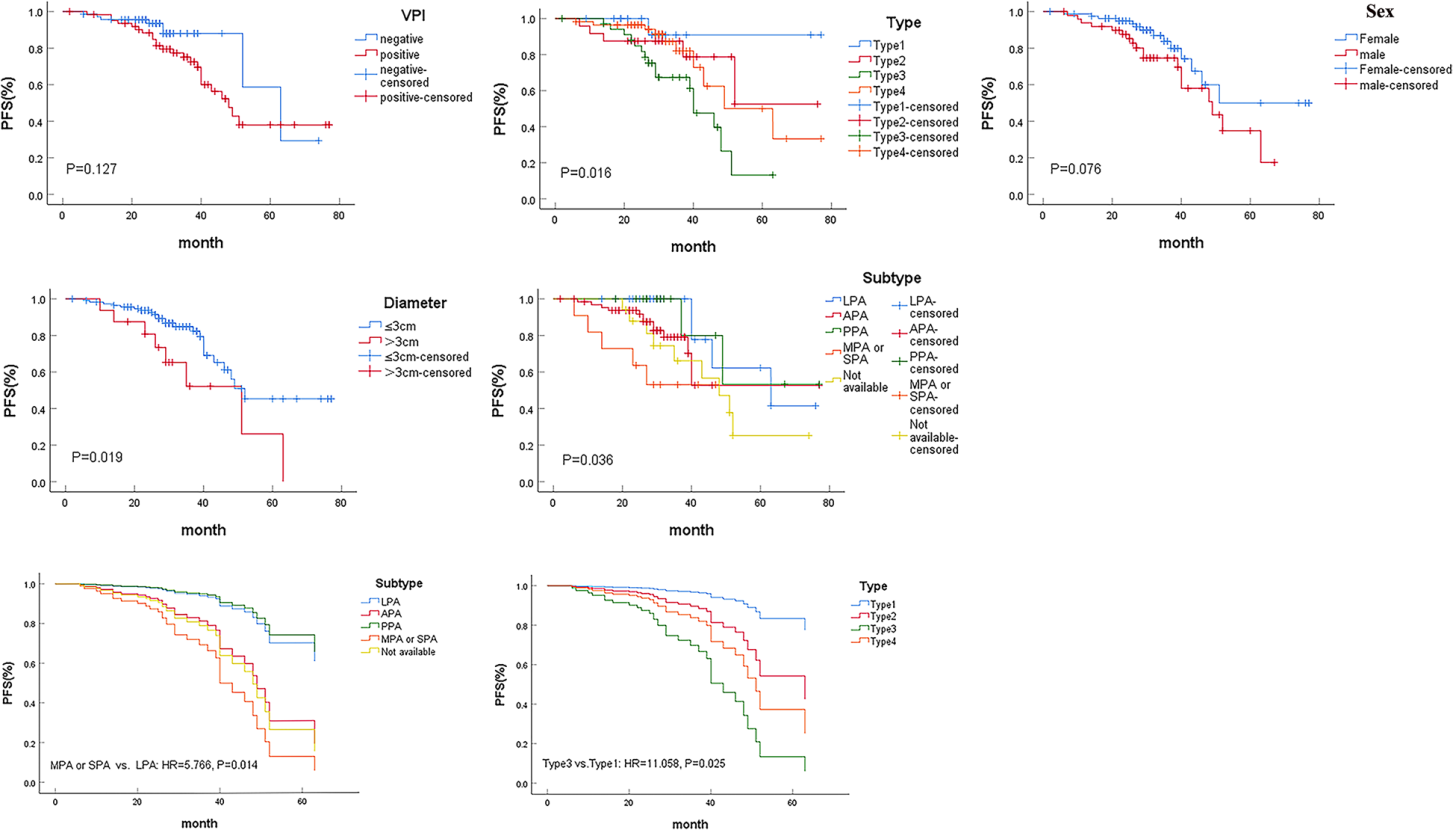
Kaplan–Meier (K-M) survival curves of variables with *P* < 0.15 and COX curves of multivariable analysis results

The results of the univariate analysis affecting tumor progression are shown in Table 3. The univariate and multivariable survival analysis curves were shown in Fig. 4. Univariate analysis showed that tumor size, histological subtype and PTs (type) were significantly associated with prognosis. Cox-proportional hazards model was further used to analyze the prognostic factors. The variables with *p* value < 0.15, such as sex, tumor size, VPI, PTs and histological subtype, were included in the analysis to exclude the mutual influence of each factor on the prognosis in univariate analysis. The Cox regression survival curve showed that micropapillary or solid histological subtype (HR = 5.766, 95% CI: 1.435–23.159, *P* = 0.014) and type 3 of PTs (HR = 11.058, 95% CI: 1.349–90.623, *P* = 0.025) were two independent risk factors for tumor progression.

**Table 3:** The results of univariate analysis affecting tumor progression in 132 patients

Variables	Progression (*n* = 32)	Progression-free (*n* = 100)	*P*
Age(year)			0.308
≤ 60	14	56	
> 60	18	44	
Sex			0.076
Male	18	32	
Female	14	68	
VPI			0.127
Yes	24	39	
No	8	61	
Largest diameter (cm)			0.019
≤ 3	24	92	
> 3	8	8	
Pathological type			0.937
MIA	1	2	
INMA	30	94	
IMA	1	4	
Histological subtype			0.036
LPA	4	19	
APA	12	54	
MPA or SPA	5	6	
PPA	2	13	
Not available	9	8	
pT stage			0.222
T1	6	55	
T2	25	44	
T3 ~ T4	1	1	
Type			0.016
1	1	16	
2	5	19	
3	16	19	
4	10	46	

*Note*. Type: different type of pleural tags

## Discussion

**Fig. 5:**
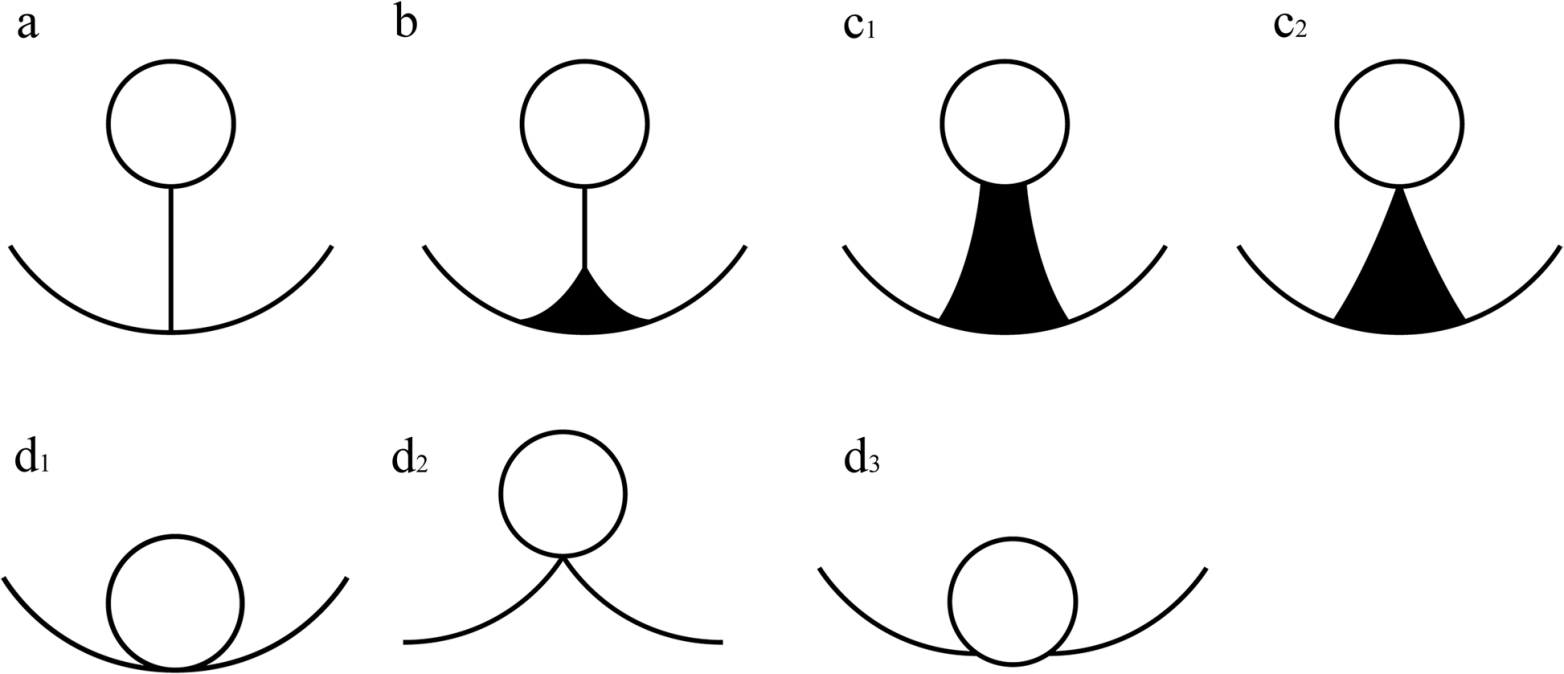
Different types of pleural tags represented by pictograms. (a) type 1, one or more linear PT; (b) type 2, one or more linear PT with soft tissue component at the pleural end; (c1) type 3, one soft tissue cord-like PT; (c2) type 3, V-shape PT; (d1, d2, d3) type 4, directly abutting, pulling and pushing the visceral pleura

Hsu et al. categorized PTs into 3 types and firstly evaluated the association between PTs and VPI of NSCLC that did not about the pleural surface. They believed that type 2 of PTs could diagnose VPI with high accuracy and specificity [10]. Yang et al. categorized PTs into nine types and found that type 3 and type 4 of PTs may be indicators for VPI [11]. Based on the studies of these two scholars, PTs were classified into four types in our study (Fig. 5). Our study found that the incidence of VPI was high in type 2 (46.88%) and type 3 (56.41%) of PTs. According to our study, type 4 of PT often suggests that there is less possibility of VPI pathologically than type 2 and type 3. Excluding severe pleural adhesion due to inflammatory diseases, chest CT combined with artificial pneumothorax is useful for the evaluation of the extension of lung cancer into the chest wall [12]. This may help us diagnose VPI of type 4 of PTs.

VPI was a significant prognostic factor independently of tumor size, histology of tumor, lymph node status, age, sex, and smoking status [5]. In several previous reports, patients with tumors exhibiting pleural invasion had worse outcomes than those without pleural invasion [13–18]. Therefore, we included VPI (*P* = 0.127) into the multivariable analysis. Our prognostic results suggested that in addition to histological subtype which was an independent risk factor for poor prognosis, type 3 of PT was also an independent risk factor for tumor progression. Although the specific mechanism of PTs affecting prognosis has not been clear, and the correlation study with gene mutation has not been reported, it still suggests to us that when this type of PT appears on CT, it should be considered whether adjuvant therapy is required after surgery.

There were some limitations in our study. Although we chose patients from three hospitals, the sample size is relatively small, so patient selection bias is inevitable, and the follow-up time is short, which may affect our results. Larger sample studies and longer follow-up are needed to validate the value of PTs in the prognosis.

At present, artificial intelligence assisted diagnosis technology has appeared, which has certain advantages in diagnosing the benign or malignant pulmonary nodules on CT and even the occurrence of VPI. However, the prognosis of different types of PTs is different. In the future, artificial intelligence combined with our prognosis study will provide help in the formulation of a comprehensive treatment plan.

## Conclusions

In conclusion, despite the limitations mentioned above, PT remains a risk factor for poor prognosis of patients with peripheral pulmonary adenocarcinoma. The presence of PTs on CT images can remind us to provide patients with a more complete surgical resection, pay attention to the postoperative pathological results and provide more reasonable treatments for the next steps.

## Data Availability

To preserve patient confidentiality, the datasets generated for this study are not publicly available, but are available upon reasonable request.

## Abbreviations

APA: Acinar predominant adenocarcinoma
CT: Computed tomography
IMA: Invasive mucinous adenocarcinoma
INMA: Invasive non-mucinous adenocarcinoma
LPA: Lepidic predominant adenocarcinoma
MIA: Minimally invasive adenocarcinoma
MPA: Micropapillary predominant adenocarcinoma
NSCLC: Non-small cell lung cancer
PPA: Papillary predominant adenocarcinoma
PT: Pleural tag
SPA: Solid predominant adenocarcinoma
TNM: Tumor node metastasis
UICC: Union for International Cancer Control
VPI: Visceral pleural invasion
